# Gastric neobladders: surgical outcomes of 91 cases using different techniques

**DOI:** 10.1590/S1677-5538.IBJU.2017.0563

**Published:** 2018

**Authors:** Aloysio Floriano de Toledo, Carlos Eduardo Bastian da Cunha, Christian Heinz Steppe, Daniel Weissbluth de Toledo, Jorge Antonio Pastro Noronha, Gustavo Carvalhal

**Affiliations:** 1 Faculdade de Medicina, Pontifícia Universidade Católica do Rio Grande do Sul - PUC, Porto Alegre, RS, Brasil

**Keywords:** Surgical Procedures, Operative, Urinary Diversion, Cystectomy

## Abstract

**Introduction:**

We report on the surgical results of a series of 91 patients who received gastric neobladders as urinary diversion after radical cystectomies performed for the treatment of muscle-invasive bladder cancers.

**Materials and Methods:**

We report on a retrospective case series of 91 patients who received gastric neobladders as urinary diversion after radical cystectomies performed for the treatment of muscle-invasive bladder cancers. Different techniques of gastric neobladders were employed from 1988 to 2013 at a university hospital in the South of Brazil.

**Results:**

Initial outcomes utilizing Leong (Antral) and Nguyen-Mitchell (Wedge) technique were unsatisfactory, yielding high pressure, low capacity reservoirs. Further developments of these techniques, with the detubularized gastric neobladder and the “spherical” gastric neobladders resulted in low pressure, high capacity reservoirs, with better surgical and urodynamic outcomes. Complication and perioperative mortality rates of our series of gastric neobladders were significantly higher than historical results of techniques using ileum or colon.

**Conclusions:**

Stomach is an exceptional option for the creation of neobladders after radical cystectomies, but due to the increased complication rates it should be reserved for specific situations (e.g., renal insufficiency, previous pelvic/abdominal radiotherapy, short bowel syndromes).

## INTRODUCTION

Neobladders are considered the gold standard among the many techniques of urinary diversion employed after radical cystectomy. Usually, ileum, right colon or sigmoid colon constitute the preferred intestinal segments utilized in these instances, but are associated with a series of complications such as malabsorption, metabolic disturbances, reservoir stones and neoplasia. Stomach has been used clinically in the construction of neobladders since the pioneering works of Sinaiko (1) in 1956 and of Leong (2), who in 1978 published their series of neobladders using the antral segment of the stomach. Later, in 1991 Nguyen and Mitchell (3) described a gastric neobladder using the gastric fundus with better results (4–6). Lockhart et al. (7) described a gastric neobladder using the gastric fundus reported in 1993 a similar technique with the addition of a segment of ileum for the creation of a composite reservoir. In 1994, Hauri et al. (8, 9) described a technique utilizing the omental bursa and the trapezoidal segment. More recently, in 2009, Wang et al. (10) reported having obtained satisfactory results with a technique of laparoscopic gastric neobladder.

Since 1988, our center has acquired an experience of 91 cases of gastric neobladders using different techniques, from a modification of Leong's technique (Antral) to Nguyen-Mitchell's technique (Wedge) and finally to what we later called the detubularized and the spherical gastric neobladders. All of the gastric neobladders were performed in patients with muscle-invasive bladder cancer. Our results show that stomach can be used in the confection of neobladders in specific situations, although in our series it was associated with a high index of surgical complications and considerable postoperative mortality (11–17). The purpose of our manuscript is to report and discuss a large series of gastric neobladders performed by a single surgeon with different techniques.

## MATERIALS AND METHODS

We report on a retrospective case series of 91 patients who received gastric neobladders as urinary diversion after radical cystectomies performed for the treatment of muscle-invasive bladder cancers from 1988 to 2013 at a university hospital in the South of Brazil. Of the 91 patients, 89 were males and 2 were females. Mean age was 72±years (33 to 81 years). All cases were performed consecutively by the same surgeon, through a variety of surgical techniques (Table-1). Surgical outcomes were accessed prospectively and through chart reviews, and entered into a computerized database.

**Table 1:** Consecutive Series of Gastric Neobladders.

Surgical technique	Cases (n)
Antral	4
Wedge	4
Detubularized	33
Spherical	50

## RESULTS

### Antral Neobladders

The first four gastric neobladders at our center were performed in 1988 according to the technique of Leong (2). Results, however, were unsatisfactory. All four surgeries resulted in high-pressure reservoirs (>50 cm H2O), with both nocturnal and diurnal enuresis. One of the patients died in the third postoperative week of sepsis, ant the other three were converted to ileal conduits.

### Wedge Neobladders

After the frustrating results with Leong technique, our group performed four cases utilizing Nguyen-Mitchell (5) technique (Wedge). Of these, only one case achieved a reservoir with an adequate capacity (close to 300 cc), while the other three had to be augmented with an ileal segment according to Lockhart's technique.

### Detubularized Gastric Neobladders

Having obtained bad results with the previously described techniques, in 1989 we developed a modification of Leong's (2) technique by performing a 50% gastrectomy, detubularizing the gastric flap by incising the lesser curvature and reconfiguring the reservoir. Gastric reconstruction was performed in a Billroth I fashion. We performed 33 neobladders with this technique, with better reservoirs (500 – 800 cc), with improved urodynamic results and with no case of postoperative hydronephrosis. Of the 33 cases, we had five postoperative deaths, two due to myocardial infarction, two due to pulmonary embolism, and one due to abdominal sepsis secondary to dehiscence of the gastro-gastric anastomosis. We observed five cases of hematuria-dysuria syndrome which were managed with proton pump inhibitors. Additionally, transient bradygastria was present in 11 patients, and treated with IV metoclopramide.

### “Spherical” Gastric Neobladders

**Figure 1:**
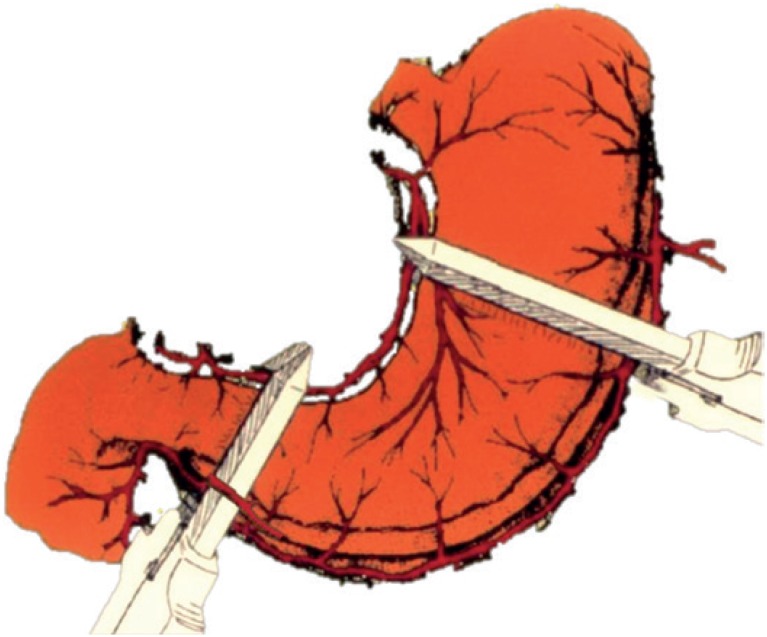
Schematic diagram of a 50% gastrectomy in the technique of the “spherical” gastric neobladder, using two 75 mm GIA staplers, with preservation of the right gastroepiploic artery.

**Figure 2:**
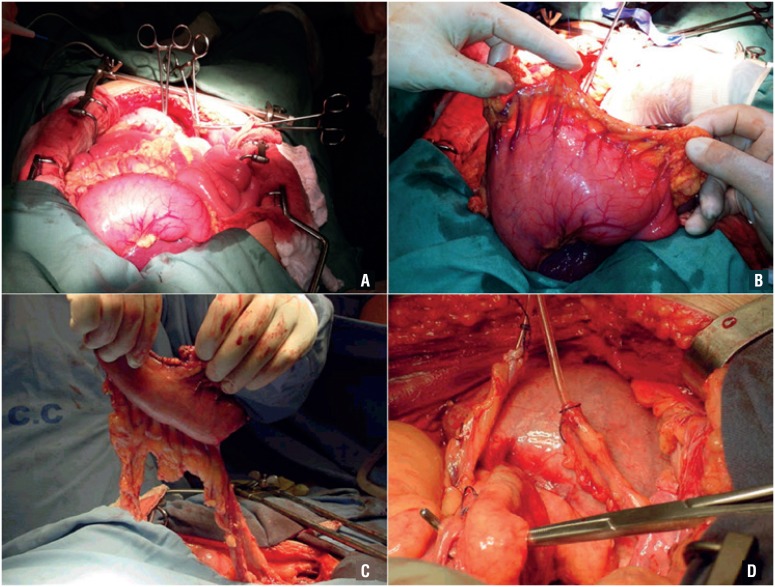
A) Evaluation of the stomach; B) Gastric reservoir with the right gastroepiploic pedicle; C) Mobilization of the gastric reservoir; D) Gastric neobladder anastomosed to the urethra, before the ureteral anastomoses, with a tension-free vascular pedicle.

Since 1994, we have performed 50 cases with our own technique of “spherical” gastric neobladders. In this technique, we use a more central portion of the stomach, and employ 75 mm GIA staplers to create an almost spherical neobladder, without the need to detubularize it (Figure-1). Such technique requires less operative time not only due to the use of staplers but also due to the fact that we perform it with two surgical teams working simultaneously; while one team prepares the gastric flap and reconstructs the stomach (Billroth I), the other team performs the radical cystectomy with pelvic lymphadenectomy. This has allowed for less hypothermia and metabolic acidosis, with mean surgical times of roughly 4.5 hours. The main steps of the confection of the “spherical” neobladder are depicted in Figure-2. Initially, the stomach is inflated with air through a nasogastric tube in order to evaluate the viability of the confection of the gastric reservoir (Figure-2A). Figure-2B shows the stapled reservoir with its vascular pedicle, produced by skeletonizing the right gastroepiploic artery up to the gastroduodenal artery. The pedicle is usually long enough to reach the pelvis (Figure-2C), and is anastomosed to the urethra after being mobilized through the transverse mesocolon and the mesentery. The ureters can be easily implanted in the anterior portion of the reservoir, through Gregoir's technique (Figure-2D).

Urodinamically, these “spherical” neobladders result in profiles similar to those obtained with the detubularized technique, with reservoirs with capacities ranging from roughly 550 cc to 800 cc (mean 675 cc). Of the 50 cases with this technique, we had eight deaths (four due to sepsis, two due to myocardial infarction, and two due to pulmonary embolism). We have treated hematuria-dysuria syndrome in nine cases, transient bradygastria in seven cases, ureteral stenosis in two, and ureteral fistula in one. Furthermore, two of our cases had to be converted into an ileal conduit due to necrosis of the neobladder. Table-2 depicts the complications observed in our 50 cases of “spherical” neobladders according to the Clavien-Dindo classification of surgical complications.

**Table 2:** Complications observed in our 50 cases of “spherical” neobladders according to the Clavien-Dindo classification of surgical complications.

Grade	I	II	IIIa	IIIb	IVa	IVb	V	Total
n	16			5			8	29
%	32			10			16	58
Bradygastria	7							
Sepsis							4	
Hematuria-dysuria	9							
Fistula/ureteral stenosis				3				
Necrosis				2				
Cardiopulmonary							4	

## DISCUSSION

The stomach is considered an exceptional option for the construction of neobladders after radical cystectomy. Traditionally, it is employed in very specific circumstances such as renal insufficiency, previous abdominal/pelvic radiotherapy and short bowel syndrome. Currently, the most utilized segment for the construction of neobladders is ileum, although some authors still prefer colon or sigmoid We report on a retrospective series of different techniques of gastric neobladders performed over a large time width, starting at a time when it was not clear which intestinal segment was best for bladder reconstruction. Stomach was being largely used for bladder augmentation in pediatric urology at the time when our series was initiated, and the indication was mainly based on the main surgeon's preference and experience. Our group has accumulated an experience of more than 25 years with different techniques of gastric neobladders as urinary diversion after radical cystectomy (11–17). After experiencing the techniques of Leong (Antral), Nguyen-Mitchell (Wedge) neobladders and detubularized gastric neobladders we developed a modification of the Leong's original technique which we named “spherical” gastric neobladder (15). This last technique, due to the ease of confection with the use of GIA staplers, and to advantages concerning capacity, urodynamic profile, and bilateral ureteral reimplantation is our preferred technique when considering stomach for a neobladder (17).

The analyses of our results deserve several considerations. Our initial experience with gastric neobladders using Antral and Wedge techniques was catastrophic.. In all cases, we ended with small reservoirs, and had to either augment or convert them to ureteroileostomies five out of eight neobladders, which led us to abandon these techniques. The technique of detubularized gastric neobladder yielded better results in terms of volume and urodynamic profile of the reservoir, but involved an increase number of suture lines and was time consuming (17). The “spherical” technique was a natural evolution of the previous attempts, and we believe is a better overall surgery, reducing the number of suture lines and decreasing surgical time (15–17).

Even with the “spherical” technique, our morbidity and mortality rates were unacceptably high and much greater than the reported mortality rates with the use of other intestinal segments, such as ileum or colon. Other groups which utilized gastric segments for bladder reconstructions after radical cystectomy did not report on perioperative mortality, although their complication rates were fairly similar to ours (2–4, 6–10, 19–22). An additional concern with surgical techniques using gastric resections is the possibility of disturbances in vitamin D and calcium metabolism, with an increased risk of vertebral fracture reported in the literature (23). We could not access this in our series since most patients did not have long-term follow-up. Likewise, we could not observe major effects associated with hypergastrinemia, such as an increased incidence of peptic disease.

Our average discharge time was between two and three weeks for most patients. This series was performed before the generalization of the enhanced recovery after surgery (ERAS) protocols, which are currently adopted at our institution. If we would perform these techniques of gastric neobladders nowadays, ERAS protocols would certainly reduce discharge time. Current ERAS protocols recommend instituting early enteral nutrition in gastric surgery patients though nasoenteral tubes or jejunostomies, which would probably reduce substantially the incidence of complications and the length of hospital stay of these patients.

We believe that our increased complication and mortality rates in the series of gastric neobladders are due to several reasons. First, the patients in our series were mostly derived from the Brazilian public health system, which often resulted in the late referral of more advanced cases, in patients with poor nutritional status and preoperative clinical conditions far from optimal. Second, we acknowledge that a limitation of these techniques with stomach is the greater distance between the gastric flap and the pelvis, creating a vascular pedicle that crosses the transverse mesocolon and the mesentery, which may lead to ischemia and necrosis, as was observed in two out of 50 cases of our “spherical” neobladders. Third, the syndrome of dysuria-hematuria is due to the contact of the acid gastric secretions and the acidic urine with the remaining urothelium in the urethra, and peculiar to the use of gastric segments in urinary diversions; although easily managed with H-2 blockers and proton pump inhibitors, we observed this phenomenon in nine out of 50 cases with the “spherical” gastric neobladders (18%) and in five out of 33 cases with the detubularized gastric neobladders (15%). Finally, we have difficulty in accessing the follow-up of many of our patients who live in distant locations from our university center, and cannot ascertain long-term result in survival and complications in the present series.

## CONCLUSIONS

We believe that stomach is an exceptional option for the creation of neobladders after radical cystectomies, but due to the increased complication rates it should be reserved for specific situations (e.g., renal insufficiency, previous pelvic/abdominal radiotherapy, short bowel syndromes).
